# Ocular surface and meibomian gland evaluation in euthyroid Graves’ ophthalmopathy

**DOI:** 10.1007/s10792-024-02919-y

**Published:** 2024-03-02

**Authors:** Kenneth Ka Hei Lai, Xulin Liao, Fatema Mohamed Ali Abdulla Aljufairi, Jake Uy Sebastian, Andre Ma, Yiu Man Wong, Cheuk Lam Lee, Wanxue Chen, Zhichao Hu, George P. M. Cheng, Clement C. Tham, Chi Pui Pang, Kelvin K. L. Chong

**Affiliations:** 1 Department of Ophthalmology and Visual Sciences, Hong Kong Eye Hospital, The Chinese University of Hong Kong, 147K Argyle Street, Kowloon, Hong Kong, Hong Kong SAR; 2 Department of Ophthalmology, Salmaniya Medical Complex, Government Hospitals, Manama, Bahrain; 3 Department of Ophthalmology, Hong Kong Sanatorium and Hospital, Hong Kong, Hong Kong SAR; 4 Department of Ophthalmology and Visual Sciences, Prince of Wales Hospital, Hong Kong, Hong Kong SAR; 5 Faculty of Medicine, The University of Hong Kong, Hong Kong, Hong Kong SAR; 6 Hong Kong Laser Eye Center, Hong Kong, Hong Kong SAR; 7 Hong Kong Eye Hospital, Hong Kong, Hong Kong SAR; 8 Department of Medicine, The University of Hong Kong, Hong Kong, China

**Keywords:** Ocular surface disease, Meibomian gland dysfunction, Euthyroid Graves’ Ophthalmopathy, Thyroid eye disease

## Abstract

**Purpose:**

Euthyroid Graves’ ophthalmology (EGO) refers to the subgroup of thyroid eye disease patients with distinct clinical presentations. This study evaluated the ocular surface and meibomian gland changes in EGO patients.

**Methods:**

A cross-sectional study was conducted at The Chinese University of Hong Kong including 34 EGO patients and 34 age-and sex- matched healthy controls. Outcome measures include anterior segment examination, keratographic and meibographic imaging.

**Results:**

Between 34 EGO patients and 34 age and sex-matched healthy controls, EGO was associated with a higher ocular surface disease index (*P* < 0.01), higher severity of meibomian gland dropout (upper: *P* < 0.001, lower: *P* < 0.00001) and higher percentage of partial blinking (*P* = 0.0036). The worse affected eyes of the EGO patients were associated with corneal staining (*P* = 0.0019), eyelid telangiectasia (*P* = 0.0009), eyelid thickening (*P* = 0.0013), eyelid irregularity (*P* = 0.0054), meibomian gland plugging (*P* < 0.00001), expressibility (*P* < 0.00001), and meibum quality (*P* < 0.00001). When the two eyes of the same EGO patient were compared, the degree of meibomian gland dropout was higher among the worse affected eyes (upper: *P* < 0.00001, and lower: *P* < 0.00001). Tear meniscus height, lipid layer thickness, and noninvasive break-up time were comparable between the two eyes of EGO patients and also between EGO patients and healthy controls. TMH was positively correlated with the degree of exophthalmos (*r* = 0.383, *P* < 0.05).

**Conclusion:**

EGO patients have more ocular surface complications and meibomian gland dropouts than healthy controls. Almost 60% of them had dry eye symptoms, but aqueous deficiency was not apparent. Further studies are warranted to clarify the mechanism of dry eye in EGO.

(249 words).

## Introduction

Thyroid eye disease (TED) is an inflammatory orbital disease that is commonly associated with autoimmune thyroid disease that can occur in both adults and pediatric patients.^1^ Euthyroid Graves’ Ophthalmopathy (EGO) is the subgroup of TED patients who present with normal thyroid function and without a history of thyroid dysfunction or antithyroid treatment.^2,3^ The prevalence of EGO in TED patients varies from 0.9% to 15.4% among different populations.^4^ In comparison with TED, EGO is associated with more males, lower clinical activity scores, and more asymmetrical presentation. It was associated with less soft tissue inflammation, bigger exophthalmos difference, and lower risk of optic neuropathy. ^6,7^ Due to the low incidence and distinct clinical characteristics with normal thyroid function examination, the diagnosis of EGO is often challenging.

Dry eye disease is a multifactorial disease involving the tears and ocular surface that results in ocular discomfort, visual disturbance, and tear film instability.^8^ It poses an impact on the quality of life if not properly treated and managed. ^9^ Ocular surface changes were found in up to 85% of TED patients and were traditionally considered as a result of exophthalmos and corneal exposure.^10–12^ Lacrimal gland expresses thyroid-stimulating hormone receptors, and in TED endocrine complications may lead to aqueous deficiency dry eye.^13^ Overexpression of pro-inflammatory cytokines was associated with the changes in ocular surface homeostasis among TED patients.^14^ Meibomian gland dysfunction results in evaporative dry eye, and both glandular dropout and thicker lipid layers were found in active and inactive TED patients.^15^ Despite the intriguing relationship, the mechanism of the development of dry eye disease in TED is not completely understood. EGO is a subtype of TED with milder clinical manifestations. The ocular surface changes of EGO have not been reported. In this study, we evaluated the ocular surface and meibomian gland changes in EGO patients.

## Methods

This is a cross-sectional study including 34 EGO patients and 34 healthy controls. The ocular surface evaluation for the EGO patients and healthy controls were performed at the Chinese University of Hong Kong between July 2022 and December 2022.^16^ The EGO patients were all managed at the Thyroid Eye Clinic at The Chinese University of Hong Kong between September 2007 and July 2021, and the healthy controls were recruited from a community eye screening program at the Chinese University of Hong Kong.^16^ All healthy controls were sex- and age-matched, without a history of systemic inflammatory disease, inflammatory disease, neoplastic disease, ophthalmic disease, and received ocular treatment including any surgery, topical eyedrops, or contact lenses in the past 12 months. This study was approved by the Institutional Review Board. Written informed consent was obtained from every individual participant, and all study procedures conformed to the tenets of the Declaration of Helsinki.

The diagnosis of TED was made by the oculoplastic surgeons based on Bartley’s criteria^17^. EGO was confirmed and classified with the support of orbital images,^18^ (computer tomography or magnetic resonance imaging), serological evidence of thyroid-related antibodies (TRAB, TSI, TPO, or TG), and histological features compatible with TAO^19^. All EGO patients must also fulfill the following criteria:

1. Normal Triiodothyronine (T3) and thyroid stimulating hormone (TSH) within 12 months after EGO onset.
2. Without a history of thyroid dysfunction.
3. Without a history of prior treatment for autoimmune thyroid disease.
4. Without radiological evidence of orbital mass.

### Ocular surface evaluation

Slip-lamp examination was performed by one of the two masked ophthalmologists on the following nine meibomian gland dysfunction items: corneal fluorescein staining, lid telangiectasia, lid margin thickness, eyelid margin irregularity, lid-parallel conjunctival folds, papillae, meibomian gland plugging, meibomian gland expressibility, and meibum quality.^20^

Oxford Scheme was used to grade corneal fluorescein staining.^21^ Lid margin telangiectasia was graded on a scale from 0 to 3: 0 = no lid margin redness or telangiectasia, 1 = redness in the lid margin, and no telangiectasia crossing the meibomian gland orifices, 2 = redness in lid margin and telangiectasia crossing meibomian gland orifices moderate telangiectasia or redness, and 3 = severe telangiectasia or redness.^22^ Lid margin thickness was graded on a scale from 0 to 2, i.e., 0 = no thickening, 1 = lid margin thickening with or without localized rounding, and 2 = lid margin thickening with diffuse rounding.^22^ Eyelid margin irregularity was graded from 0 to 2: 0 = no irregularity, 1 = fewer than 3 lid margin irregularities and shallow notching, and 2 = three or more lid margin irregularities or deep notching.^22^ Lid-parallel bulbar conjunctival fold was graded on a scale from 0 to 3: 0 = without fold, 1 = single small fold, 2 = more than two folds, and not higher than the tear meniscus height, 3 = multiple folds and higher than the tear meniscus.^23^ Papillae were graded using the papillae-limbal corneal epithelial score.^24^ Meibomian gland plugging was assessed using the scale from 0 to 3: 0 = no plugging, 1 = fewer than three pluggings of gland orifices, 2 = three or more pluggings of gland orifices with a distribution of less than half of the full length of the eyelid, and 3 = three or more pluggings of glands orifices with a distribution of half or more of the full length of the lid.^21^ Meibomian gland expressibility was graded from 0 to 3 based on the central 8 meibomian glands of the lower eyelid: 0 = all glands expressible, 1 = 3–4 glands expressible, 2 = 1–2 glands expressible, and 3 = no gland expressible.^25^ Meibum quality was graded on a scale of 3: 0 = clear, 1 = cloudy, 2 = cloudy with debris, and 3 = inspissated, toothpaste-like. ^25^

Subjective dry eye symptom was measured using the Ocular Surface Disease Index (OSDI) 12-item questionnaire.^26^ Baseline tear production was measured by Schirmer’s test without topical anesthetic.^27^ The noninvasive tear film break-up time (NITBUT) and tear meniscus height (TMH) were measured using a corneal topographer with a built-in real-time keratometer (Oculus1 Keratograph 5 M, Oculus Inc, Arlington, WA*).* Lipid layer thickness (LLT), partial blinking (PB) rates, and meibography were obtained using the tear interferometer *(*LipiView1, TearScience Inc, Morrisville, NC). The meibomian gland dropout was measured from the meibograph using the ImageJ software. The area covered by tarsal conjunctiva and meibomian glands dropout was segmented using a freehand tool by two independently trained masked observers, and the average was used for data analysis. The degree of meibomian gland dropout was measured by dividing the meibomian gland dropout area by the total area of the tarsal conjunctiva.

Numerical results were presented as mean ± standard deviation (SD) and range unless otherwise stated. The comparison of ocular surface evaluation between EGO patients and healthy controls. The worse affected and less affected eyes of EGO patients were measured using the paired T test. The worse affected eye was confirmed by 1 ophthalmologist by reviewing the clinical orbital parameters and orbital imaging. All statistical analyses were performed using SPSS statistical software package (Windows version 24.0; IBM Corp., Armonk, NY).

## Results

Totally 34 ethnic Han Chinese EGO (17 males, 17 females) patients and 34 age and sex-matched (17 males) healthy controls. All the patients did not receive any treatment for EGO before presenting to the thyroid eye clinic. The age on ocular surface examination was 57.3 ± 13.9 and 57.3 ± 11.1 years for EGO patients and controls. The presenting clinical activity score (CAS) was 1.1 ± 1.1, and 15 (44%) patients presented unilaterally. The clinical information of EGO patients is summarized in Table 1.

**Table 1:** Clinical information of euthyroid Graves’ ophthalmology (EGO) patients on presentation

	EGO patients, *n* = 34
Age of EGO onset (years)	55.3 ± 17.1
Current/Ex-Smoker	13 (40%)
Unilateral presentation	15 (44%)
Clinical activity score (CAS)	1.1 ± 1.1
Marginal reflex distance (MRD) 1 (mm)	4.4 ± 1.7
Marginal reflex distance (MRD) 2 (mm)	4.7 ± 2.0
Exophthalmos	18.1 ± 3.9
Extraocular motility (EOMy)	1.5 ± 1.4
Diplopia	0.9 ± 1.6
Dysthyroid optic neuropathy (DON)	3 (9%)
Intravenous methylprednisolone (IVMP)	13 (38%)
Orbital radiotherapy (ORT)	10 (29%)
Surgical decompression	3 (9%)

We compared the worse affect eyes of EGO patients with the right eyes of controls, the degrees of corneal staining (*P* = 0.0019), eyelid margin changes including telangiectasia (*P* = 0.0009), thickening (*P* = 0.0013), irregularity (*P* = 0.0054), meibomian gland plugging(*P* < 0.00001), expressibility(*P* < 0.00001), and meibum quality (*P* < 0.00001) were associated with the worse affected eyes of EGO patients** (**Table 2**).** EGO eyes were found to have a higher degree of meibomian gland dropout (upper: *P* < 0.001, and lower: < 0.00001). EGO patients had higher scores on the ocular surface disease index (OSDI) (*P* < 0.01). EGO patients had a higher percentage of partial blinking (*P* = 0.0036) **(**Table 3**).** We also compared the worse affected eyes with the better eyes among the 34 EGO patients. Meibomian gland dropout was more severe in the worst affected eyes (upper: *P* < 0.00001, and lower: *P* < 0.00001) **(**Table 4**).** TMH was positively correlated with the degree of exophthalmos (r = 0.383, *P* < 0.05**) (**Table 5**)**.

**Table 2:** Comparison of anterior segment features between EGO patients and healthy controls

	EGO patients	Healthy controls	*P* value
Number of eyes	34	34	Not applicable
Corneal staining	1.2 ± 1.3	0.9 ± 0.6	0.029
Telangiectasia	1.4 ± 1.4	0.7 ± 0.6	0.000087
Eyelid margin thickening	1.2 ± 1.2	0.9 ± 0.7	0.029
Eyelid margin irregularity	0.6 ± 0.6	0.3 ± 0.3	0.042
Lid-parallel conjunctival folds	0.6 ± 0.6	0.4 ± 0.5	0.83
Papillae	0.6 ± 0.6	0.5 ± 0.6	0.30
Meibomian gland plugging	1.6 ± 1.6	0.7 ± 0.8	0.000024
Meibomian gland expressibility	1.7 ± 1.7	0.8 ± 0.8	0.000097
Meibum quality	2.0 ± 1.8	0.9 ± 0.8	0.000093

*EGO*  Euthyroid Graves’ ophthalmology

**Table 3:** The comparison of ocular surface evaluation between EGO patients and healthy controls

	EGO patients	Healthy controls	*P* value
Number of eyes:	34	34	N/A
Tear meniscus height (TMH) (mm)	0.3 ± 0.1	0.3 ± 1.1	0.39
NITBUT average (seconds)	14.2 ± 4.8	15.1 ± 4.7	0.31
Schirmer’s test (mm)	13.6 ± 13.6	10.2 ± 10.1	0.080
Ocular surface disease index (OSDI)	24.0 ± 22.9	12.3 ± 11.7	0.010
Upper MG dropout (%)	38.7 ± 14.0	15.7 ± 7.3	< 0.0001
Lower MG dropout (%)	31.0 ± 10.4	23.8 ± 9.8	< 0.00001
Lipid layer thickness (LLT) average (nm)	62.3 ± 18.1	63.7 ± 19.0	0.40
Partial blinking (%)	80.0 ±	65.2 ±	0.036

*EGO* Euthyroid Graves’ ophthalmology, *MG * Meibomian glands

**Table 4:** Comparison of ocular surface evaluation between the 2 eyes in EGO patients

	Worse eye	Better eye	*P* value
Number of eyes	34	34	N/A
Tear meniscus height TMH (mm)	0.3 ± 0.1	0.3 ± 0.1	0.47
NITBUT average (seconds)	14.2 ± 4.8	13.1 ± 4.5	0.14
Schirmer’s test (ST) (mm)	13.6 ± 13.6	12.6 ± 9.3	0.33
Upper MG dropout (%)	38.7 ± 14.0	16.2 ± 7.8	< 0.00001
Lower MG dropout (%)	31 ± 10.4	21.5 ± 8.9	< 0.00001
Lipid layer thickness (LLT) average (nm)	62.3 ± 18.1	69.9 ± 18.9	0.079

*EGO*  Euthyroid Graves’ ophthalmology, *MG*  Meibomian glands

**Table 5:** The correlations between EGO parameters with dry eye parameters (worse eye)

	UL MG dropout		LL MG dropout		NITBUT		LLT		TMH		ST	
	*R*	*P*	*r*	*P*	*r*	*P*	*r*	*P*	*r*	*P*	*r*	*P*
*Age of EGO onset*	0.126	0.476	0.267	0.127	0.111	0.532	0.0401	0.822	0.187	0.289	0.235	0.181
*Presenting clinical activity score*	-0.140	0.430	-0.0252	0.151	0.041	0.818	0,164	0.354	0.031	0.861	0.0956	0.591
*Marginal reflex disease 1*	-0.0376	0.835	0.194	0.272	-0.221	0.209	-0.0439	0.809	-0.313	0.0715	-0.132	0.457
*Extraocular motility*	0.0489	0.784	-0.139	0.433	0.286	0.101	0.0518	0.771	0.0548	0.758	0.256	0.144
*Exophthalmos*	-0.135	-0.447	-0.123	0.488	-0.156	0.378	0.0073	0.967	0.383	0.0253*	0.229	0.193

*EGO*  Euthyroid Graves’ ophthalmology, *LL*  Lower lid, *LLT * Lipid layer thickness, *MG*  Meibomian glands, *ST*  Schirmer test, *TMH*  Tear meniscus height, *UL* ds Upper lid

*P < 0.05

## Discussion

In this cross-sectional study, meibomian gland dropouts were more severe in both the upper and lower eyelid of EGO patients when compared to controls. Notably, the degree of the dropout was more severe in the worse affected eyes in EGO patients. Clinical signs of meibomian gland dysfunction were found associated with EGO patients. EGO patients had a higher percentage of partial blinking. TMH was positively correlated with the degree of exophthalmos. EGO patients were associated with a greater degree of dry eye symptoms, and up to 58% score over 12 on the OSDI questionnaire.

Dry eye disease is the most prevalent ocular surface complication. It is long known to have a close relationship with TED, and ocular surface changes were reported in up to 85% of patients.^8,10^ Our study found 58% of EGO patients had at least mild ocular surface disease according to the OSDI questionnaire, and they had a higher degree of partial blinking. A normal blinking motion protects the ocular surface and serves as a pumping force to secrete the meibum to form the lipid tear layer.^28^ Chronic incomplete blinking may result from eyelid retraction and exophthalmos which can result in obstructive meibomian gland obstruction. Park et al. compared TED-related dry eye disease patients and non-thyroidal dry eye patients and showed that both incomplete blinking and meibomian gland dropout were associated in TED patients.^12^ One explanation for the normal lipid layer thickness despite an increased meibomian gland dropout was the compensatory effect as the meibomian glands may secrete meibum at an enhanced level to maintain the lipid layer.^29,30^ It is also possible that the remaining meibomian glands were sufficient to secrete meibum into the tear film. ^31^

Meibomian gland dysfunction is a common and multifactorial chronic ocular surface condition. Wang et al. reported meibomian gland dropout in both active and inactive TED patients.^32^ In 53 active Thai TED patients, there was a higher meibomian gland dropout than healthy controls, and the area of dropout was correlated with euthyroid status and lagophthalmos.^33^ In a study on 19 Japanese TED patients, all had obstructive meibomian gland dysfunction.^34^ The mechanism of meibomian gland structural changes in TED is complex and multifactorial. Chronic eyelid inflammation may destroy the glandular epithelium, and mechanical compression from soft tissue expansion may result in meibomian gland atrophy.^15^ In this study on Chinese EGO patients, up to 44% of them initially presented meibomian gland atrophy unilaterally. We also found that the degree of meibomian gland dropout was higher in those worst affected eyes, which may suggest a relationship between the degree of dropout and EGO disease severity.

The lacrimal gland expresses the receptors of thyroid-stimulating hormone, which may contribute to TED-related dry eye disease in those with lacrimal gland impairment.^35^ Kashkouli et al. reported that in 38 Iranian moderate to severe TED patients, there was an aqueous deficient type of dry eye with reduced tear break-up time and Schirmer test.^36^ Recently, we found occurrence of evaporative dry eye disease in Chinese TED patients.^37^ Huang et al. investigated the tear samples of Chinese TED patients and found the concentration of interleukin 1β, 6, and 8 was found higher in TED patients than controls, and TED patients were associated with a short tear break-up time and Schirmer test.^14^ On the contrary, the Chinese EGO patients in our study did not show any difference in tear meniscus height noninvasive tear break-up time and Schirmer tear, clearly indicating absence of aqueous deficient component in EGO eyes.

EGO is a subgroup of TED with distinct clinical features. EGO is more prevalent in males, associated with lower disease activity, and more asymmetrical.^5^ The activation of thyroid-stimulating hormone receptors induces the pathogenesis of both Graves’ disease and TED. EGO presents with a normal thyroid function, and the targets are tissue cells in the eye and the orbit. The absence of aqueous deficiency in our study indicates a specific pattern of ocular adnexal targets between TED and EGO patients.^37^

Exophthalmos in TED patients is related to hyaluronan deposition, leading to extraocular muscle (EOM) enlargement, mononuclear infiltrations, and orbital soft tissue expansion behind the eyeball.^38^ Exophthalmos can impair the blinking mechanism and incomplete eyelid closure in those with severe diseases. Our study showed a positive correlation between the TMH and exophthalmos level. This could be explained by the excess aqueous production as the body attempts to compensate for the poor ocular surface. In addition, EGO patients with severe exophthalmos can also affect the tear drainage system, which can lead to an accumulation of tear at the lower eyelid margin.

There are several limitations in the current study. First, the number of EGO patients included was relatively small.^39^ Second, conditions that may affect the ocular surface condition such as psychiatric disease, poor sleeping quality, and computer vision syndrome were not assessed in our EGO patients and healthy control. Third, this is a cross-sectional study without follow-up data. Forth, the associations that may affect the degree of ocular surface parameters such as lacrimal gland dimension, corneal sensation, and corneal biomechanics were not evaluated. Fifth, proportion of the EGO patients have received treatment such as orbital radiotherapy and orbital surgery that may affect the ocular surface. ^40^ Our data serves as a reference for future prospective cohorts or clinical trials to study the dry eye characteristics in EGO patients.

In summary, up to 58% of EGO patients had at least mild dry eye symptoms. Both meibomian gland dropouts and partial blinking were associated with EGO patients. Aqueous tear deficiency dry eye is absent in EGO patients. Early treatment should be commenced for EGO patients with unstable tear films, especially those with more severe disease severity. Moreover, the mechanism of dry eye disease in EGO should be further explored.

## Abbreviations

EGO: Euthyroid Graves’ ophthalmopathy
HC: Healthy controls
IRB: Institutional Review Board
LLT: Lipid layer thickness
MGD: Meibomian gland dysfunction
Mm: Millimeter
N: Number of
nm: Nanometer
N/A: Not applicable
ORT: Orbital radiotherapy
OSDI: Ocular surface disease index
PB: Partial blinking
S: Seconds
SD: Standard deviation
SSA: Steroid-sparing agents
ST: Schirmer’s test
TED: Thyroid eye disease
TMH: Tear meniscus height
